# Seminal Plasma Metabolomic Profiling Reveals Key Metabolic Signatures Linked to Spermatogenic Potential in Non-Obstructive Azoospermia with Cryptorchidism

**DOI:** 10.3390/metabo16020147

**Published:** 2026-02-23

**Authors:** Jianxing Cheng, Yanlin Tang, Qiancheng Zhao, Jiaming Weng, Zishui Fang, Yanan Qi, Hui Jiang, Zhe Zhang

**Affiliations:** 1 Department of Urology, Peking University Third Hospital, Beijing 100191, China; 2 Department of Reproductive Medicine Center, Peking University Third Hospital, Beijing 100191, China; 3 Department of Urology, Institute of Urology, Peking University First Hospital, Beijing 100134, China

**Keywords:** cryptorchidism, azoospermia, seminal plasma, metabolomics, surgical sperm retrieval

## Abstract

Background/Objectives: Cryptorchidism is a common cause of male infertility and often results in azoospermia. However, the metabolic perturbations underlying cryptorchidism complicated with azoospermia and their association with surgical sperm retrieval outcomes remain poorly defined. Methods: A total of 35 patients with cryptorchidism and azoospermia, as well as 40 controls with normal semen parameters, were enrolled in the study. Seminal plasma samples from all participants were subjected to metabolomic analysis. Additionally, some patients underwent micro-TESE; the association between metabolomic features and the success or failure of surgical sperm retrieval was further analyzed. Results: A total of 931 differential metabolites were identified between patients and controls, primarily enriched in lipid metabolism and amino acid metabolism pathways. Lipid metabolites were broadly downregulated in patients, while several inflammation-related metabolites, including Prostaglandin E2, were upregulated. Routine clinical parameters showed no significant differences between patients with successful and failed micro-TESE. However, metabolomic profiles effectively distinguished these two subgroups. These differential metabolites between the two subgroups were mainly involved in three key pathways: phenylalanine–tyrosine–tryptophan biosynthesis, aminoacyl-tRNA biosynthesis, and folate biosynthesis. Most metabolites in the first two pathways were downregulated in the successful retrieval group, while those in the folate biosynthesis pathway showed the opposite regulatory trend. Four metabolites, including Leucine, 7,8-Dihydroneopterin, L-Tyrosine and Pterin, exhibited robust predictive value for micro-TESE outcomes. Conclusions: This study reveals distinct metabolic signatures in patients of cryptorchidism with azoospermia. The identified metabolic biomarkers provide valuable references for clinical decision-making regarding micro-TESE, facilitating a personalized assessment of sperm retrieval feasibility.

## 1. Introduction

Cryptorchidism, or undescended testis, is defined as a unilateral or bilateral absence of the testes from the scrotum due to incomplete prenatal testicular descent. It is one of the most common congenital malformations of the male reproductive system [1]. Reports indicate that the incidence of cryptorchidism ranges from 1.6% to 9.0% in full-term male infants, and this proportion can be as high as 30% in premature infants [2]. Although 70% to 80% of affected children experience spontaneous testicular descent within one year after birth, untreated or persistently undescended testicles can lead to a series of reproductive and systemic complications, including infertility, testicular malignancy, and hormonal dysfunction [3,4]. Studies have shown that men with a history of cryptorchidism, even if they undergo orchidopexy at 12–18 months of age, have a 2–5-fold higher risk of spermatogenic dysfunction, with azoospermia occurring in 10–15% of unilateral and up to 40–50% of bilateral cases [5,6].

The development of cryptorchidism with azoospermia is the result of combined damage to testicular development caused by multiple prenatal and postnatal factors. During the prenatal stage, abnormal gubernaculum migration and hormonal imbalance can disrupt the process of testicular descent and the proliferation of primordial germ cells [5,7]. In the postnatal stage, the undescended testicles are exposed to the high-temperature environment of the abdominal cavity or inguinal region, which induces oxidative stress and impairs the maturation of germ cells [8,9]. In our previous research, we analyzed the pathological changes in the testicular tissue of patients with cryptorchidism with azoospermia through single-cell RNA-seq analysis, and found that the impairment of spermatogenesis in these patients is mainly associated with dysfunction in the self-renewal and differentiation of spermatogonia [10]. Despite the progress our study has made in understanding the pathophysiological mechanisms of cryptorchidism-related infertility, the molecular mechanisms underlying spermatogenic dysfunction remain incompletely clarified.

Clinically, microdissection testicular sperm extraction (micro-TESE) is the primary therapeutic option for those with cryptorchidism with azoospermia seeking biological offspring [11]. Once viable sperm are identified and retrieved from the testes, patients can achieve biological parenthood with the aid of Assisted Reproductive Technology [12]. However, the sperm retrieval rate of micro-TESE is only 30~60% [13]. Currently, predicting micro-TESE outcomes remains challenging, as traditional clinical indicators (such as hormone levels and testicular volume) lack sufficient sensitivity and specificity. For example, follicle-stimulating hormone levels are often elevated in cryptorchidism with azoospermia, but they cannot effectively distinguish between patients with residual spermatogenic function and those with complete spermatogenic arrest in the testes [14,15]. Moreover, invasive procedures such as testicular biopsy are associated with inherent risks, including testicular scarring and further iatrogenic damage to the remaining functional spermatogenic tissue [16]. In contrast, semen integrates signals from multiple reproductive organs and serves as an ideal sample for investigating the systemic and local metabolic consequences of testicular damage caused by cryptorchidism [17]. Therefore, there is an urgent need to identify reliable biomarkers for predicting sperm retrieval outcomes and elucidating the pathogenic mechanisms of cryptorchidism with azoospermia.

As a core component of systems biology, metabolomics can comprehensively profile small-molecule metabolites in biological samples, reflecting the downstream effects of genetic, epigenetic, and environmental perturbations [18]. In recent years, the application of metabolomics in reproductive health research has provided valuable insights into decoding the biochemical factors affecting male fertility [19,20]. Seminal plasma, as the immediate microenvironment for sperm survival and function, contains a variety of metabolites involved in sperm membrane formation, energy metabolism, and oxidative stress defense. Previous metabolomic studies have identified metabolic abnormalities in the seminal plasma of infertile men, such as the dysregulation of lipid metabolism and amino acid metabolism [21]. However, studies focusing on the specific metabolic signatures of cryptorchidism with azoospermia and their association with micro-TESE outcomes remain scarce.

The present study aims to systematically characterize the seminal plasma profiles of patients with cryptorchidism and azoospermia through untargeted metabolomic profiling and to identify key metabolic pathways involved in the pathogenesis of cryptorchidism with azoospermia. Furthermore, we compare the metabolic differences between patients with successful and failed micro-TESE to screen potential biomarkers predictive of sperm retrieval. These findings may contribute to a better understanding of the biological mechanisms underlying cryptorchidism with azoospermia and provide a basis for improving diagnostic and therapeutic strategies in affected patients.

## 2. Materials and Methods

### 2.1. Study Population

The samples analyzed in this study were obtained from the Andrology Laboratory of Peking University Third Hospital between October 2023 and July 2024. This study protocol has been reviewed and approved by the Institutional Review Board (IRB) of Peking University Third Hospital (Approval Number: [IRB00006761-M2022692]). The researchers have fully informed all patients of the detailed information about this research project and obtained their written informed consent for participation. The inclusion and exclusion criteria for the participants are as follows:

Cryptorchidism with azoospermia: (1) Having a history of unilateral or bilateral cryptorchidism and having undergone orchidopexy; (2) Being diagnosed with azoospermia through at least 3 semen analyses; (3) Having obstructive etiologies excluded via ultrasound examination (e.g., history of vasectomy, epididymal obstruction); (4) Having a normal chromosomal karyotype (46, XY) and no Y-chromosome microdeletions (confirmed by multiplex polymerase chain reaction targeting the azoospermia factor (AZF) a/b/c regions).

Control group: Fertile males with age matching (22–40 years old), who must meet the following criteria: (1) Normal semen parameters (in accordance with the WHO 2021 standards: sperm concentration ≥ 15 × 10^6^/mL, progressive motility sperm percentage ≥ 32%, normal morphology sperm percentage ≥ 4%); (2) No history of reproductive system diseases (e.g., orchitis, varicocele), no history of endocrine diseases (e.g., hypogonadism), and no exposure to reproductive toxic substances (e.g., heavy metals, chemotherapeutic drugs) within the past 6 months.

Before sample preparation, andrological evaluation was performed for all the participants. FSH, LH, PRL, E2 and testosterone serum levels were checked in the same laboratory (using electrochemiluminescence (ECL) immunoassays) at the Department of Reproductive Medicine Center, Peking University Third Hospital. Physical exams have been performed to make sure that all the patients have vas deferens. In addition, testicular volume has been measured with an orchidometer. Preoperative ultrasound examinations were also performed in some patients to determine the location and size of the testicles. Patients’ data, including history, physical examination, hormonal assessment, karyotypes, AZF microdeletions, biopsy results, and their sperm retrieval outcomes, were extracted from medical files. We also assessed key potential infertility risk factors (including smoking, alcohol consumption, drug abuse, and infectious history) and excluded all ineligible samples.

### 2.2. Sample Preparation

All study participants were required to abstain from sexual activity for 2–7 days prior to sample collection. Semen samples were collected via masturbation into sterile containers free of DNA/RNA contamination, and then incubated at 37 °C for 30 min to ensure complete liquefaction. Semen analysis was performed in accordance with the Fifth Edition of Semen Analysis Standards formulated by the World Health Organization (WHO) [22]. The liquefied semen was centrifuged at 3000× *g* and 4 °C for 15 min to separate the seminal plasma. The supernatant (seminal plasma) was aliquoted into sterile test tubes, immediately frozen in liquid nitrogen, and stored at −80 °C until analysis. Samples with obvious contamination (e.g., blood-stained) or incomplete liquefaction were excluded. To remove proteins, a 100 µL sample was thoroughly mixed with 400 µL of cold methanol acetonitrile (*v*/*v*, 1:1) via vortexing. And then the mixture was processed with sonication for 1 h in ice baths. The mixture was then incubated at −20 °C for 1 h and centrifuged at 4 °C for 20 min at a speed of 14,000 g. The supernatants were then harvested and dried under vacuum LC–MS analysis.

### 2.3. UHPLC–MS/MS and Analysis

Metabolomics profiling was analyzed using a UPLC–ESI-Q-Orbitrap-MS system (UHPLC, Shimadzu Nexera X2 LC-30AD, Shimadzu, Kyoto, Japan) coupled with Q-Exactive Plus (Thermo Scientific, San Jose, CA, USA). For liquid chromatography (LC) separation, samples were analyzed using an ACQUITY UPLC^®^ HSS T3 column (2.1 × 100 mm, 1.8 μm) (Waters, Milford, MA, USA). The flow rate was 0.3 mL/min and the mobile phase contained A: 0.1% FA in water and B: 100% acetonitrile (ACN). The gradient was 0% buffer B for 2 min and was linearly increased to 48% over 4 min, and then up to 100% over 4 min, where it was maintained for 2 min. It was then decreased to 0% buffer B in 0.1 min, with a 3 min re-equilibration period employed. The electrospray ionization (ESI) with positive mode and negative mode were applied for MS data acquisition separately. The HESI source conditions were set as follows: Spray Voltage: 3.8 kv (positive) and 3.2 kv (negative); Capillary Temperature: 320 °C; Sheath Gas (nitrogen) flow: 30 arb (arbitrary units); Aux Gas flow: 5 arb; Probe Heater Temp: 350 °C; S-Lens RF Level: 50. The instrument was set to acquire over the *m*/*z* range 70–1050 Da for full MS. The full MS scans were acquired at a resolution of 70,000 at *m*/*z* 200, and 17,500 at *m*/*z* 200 for MS/MS scan. The maximum injection time was set to 100 ms for MS and 50 ms for MS/MS. The isolation window for MS2 was set to 2 *m*/*z* and the normalized collision energy (stepped) was set as 20, 30 and 40 for fragmentation.

#### 2.3.1. Data Preprocessing and Filtering

The raw MS data were processed using MS-DIAL for peak alignment, retention time correction and peak area extraction. The metabolites were identified by accuracy mass (mass tolerance < 10 ppm) and MS/MS data (mass tolerance < 0.02 Da), which were matched with HMDB, massbank, other public databases and our self-built metabolite standard library. In the extracted-ion features, only the variables having more than 50% of the nonzero measurement values in at least one group were kept.

#### 2.3.2. Multivariate Statistical Analysis

R (version: 4.0.3) and R packages were used for all multivariate data analyses and modeling. Data were mean-centered using Pareto scaling. Models were built on principal component analysis (PCA), orthogonal partial least-square discriminant analysis (PLS-DA) and partial least-square discriminant analysis (OPLS-DA). All the models evaluated were tested for overfitting using permutation tests. The descriptive performance of the models was determined by R2X (cumulative) (perfect model: R2X (cum) = 1) and R2Y (cumulative) (perfect model: R2Y (cum) = 1) values, while their prediction performance was measured by Q2 (cumulative) (perfect model: Q2 (cum) = 1) and a permutation test (*n* = 200). The permuted model should not be able to predict classes: R2 and Q2 values at the Y-axis intercept must be lower than those of Q2 and the R2 of the non-permuted model. OPLS-DA allowed the determination of discriminating metabolites using the variable importance in projection (VIP). The VIP score value indicates the contribution of a variable to the discrimination between all the classes of samples. Mathematically, these scores are calculated for each variable as a weighted sum of squares of PLS weights. The mean VIP value is 1, and usually VIP values over 1 are considered as significant. A high score is in agreement with a strong discriminatory ability and thus constitutes a criterion for the selection of biomarkers.

The discriminating metabolites were obtained using a statistically significant threshold of variable influence on projection (VIP) values obtained from the OPLS-DA model and two-tailed Student’s *t* test (*p* value) on the normalized raw data at univariate analysis level. The *p* value was calculated by one-way analysis of variance (ANOVA) for multiple groups analysis. Metabolites with VIP values greater than 1.0 and *p* value less than 0.05 were considered to be statistically significant metabolites. Fold change was calculated as the logarithm of the average mass response (area) ratio between two arbitrary classes. On the other side, the identified differential metabolites were used to perform cluster analyses with the R package.

To identify the perturbed biological pathways, the differential metabolite data were subjected to KEGG pathway analysis using the KEGG database (http://www.kegg.jp, accessed on 1 August 2025). KEGG enrichment analyses were carried out with the hypergoemetric test, and FDR correction for multiple testing was performed. Enriched KEGG pathways were nominally statistically significant at the *p* < 0.05 level. Receiver operating characteristic (ROC) curves were plotted using R software (v4.2.0) to evaluate the diagnostic performance of differential metabolites.

### 2.4. Statistical Analysis

Quantitative data were expressed as mean ± standard deviation (SD) or median (interquartile range) based on normality. The primary statistical method used for comparing metabolic differences between two independent groups was the two-tailed Student’s *t* test. Metabolites with a VIP value > 1.0 and a *p* value < 0.05 were defined as significantly differential metabolites between the two groups. A prediction model for the success rate was generated on the basis of the logistic regression analysis. As detailed in the figure legends, this test was applied for all group comparisons presented.

## 3. Results

### 3.1. Study Population Characteristics

A total of 75 participants were enrolled in this study, including 35 cryptorchidism with azoospermia patients and 40 men with normal semen parameters who served as controls. In the cryptorchidism group, 29 cases (82.9%) had bilateral cryptorchidism and 6 cases (17.1%) had unilateral involvement. All participants in the control group exhibited normal semen parameters (sperm concentration ≥ 15 × 10^6^/mL, progressive motility ≥ 32%) and had no history of reproductive system diseases, as confirmed by physical examination and semen analysis. The demographic and clinical characteristics of the participants are presented in Table 1.

The mean ages of the cryptorchidism group and the control group were 32.0 ± 4.4 years and 30.5 ± 4.9 years, respectively, with no statistically significant difference (*p* > 0.05). There was also no significant difference in body mass index (BMI) between the two groups (*p* = 0.70). In contrast, the testicular volume of the cryptorchidism group was significantly smaller than that of the control group for both the left testis (8.4 ± 4.3 mL, *p* < 0.01) and the right testis (9.1 ± 5.6 mL, *p* < 0.01). Hormone level detection results showed that the levels of FSH and LH in the cryptorchidism group were significantly higher than those in the control group (*p* < 0.01), while there were no significant differences in the levels of prolactin (PRL), testosterone, and estradiol (E2) between the cryptorchidism group and the control group (all *p* > 0.05).

### 3.2. Seminal Plasma Metabolic Characteristics of Cryptorchidism with Azoospermia

**Figure 1:**
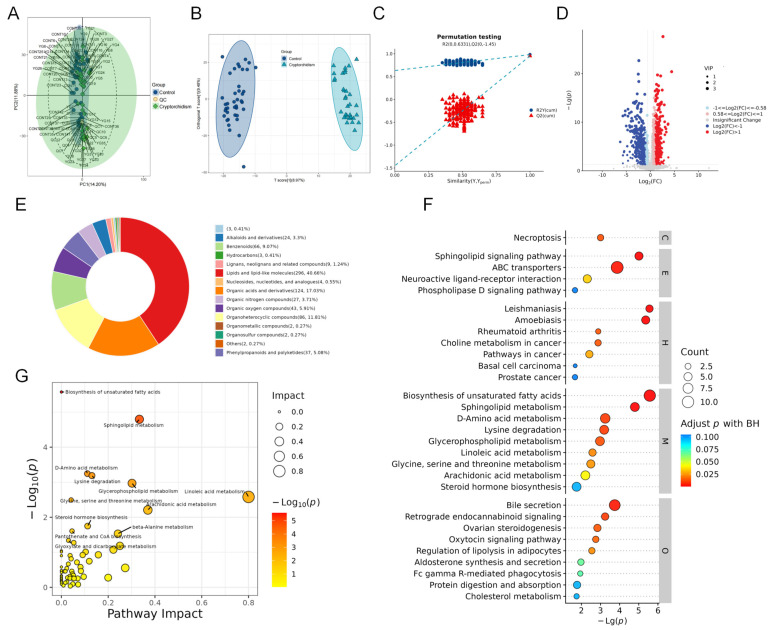
Seminal plasma metabolic characteristics of cryptorchidism with azoospermia. (**A**) PCA score plot of experimental samples and quality control (QC) samples. (**B**) OPLS−DA score plot of seminal plasma metabolomic profiles from patients with cryptorchidism (*n* = 35) and control (*n* = 40). (**C**) Permutation test plot of OPLS-DA. (**D**) Volcano plot of significantly altered metabolites, where colors correspond to fold change (FC) and the size of dots corresponds to Variable Importance in Projection (VIP) values from the OPLS−DA model. (**E**) Differential metabolites classification ring chart (HMDB Super Class). (**F**) KEGG pathway enrichment bubble plot of differential metabolites (Top 30 Pathways). (**G**) Pathway Impact Plot of KEGG (Out degree centrality). Abbreviations: VIP, variable importance in projection (from OPLS−DA).

Comprehensive metabolic profiling was performed on all samples to characterize the metabolic profiling of seminal plasma in cryptorchidism with azoospermia. After the data normalization of the peaks extracted from all experimental samples and quality control (QC) samples, principal component analysis (PCA) was performed (Figure 1A). An orthogonal partial least-squares discriminant analysis (OPLS-DA) model was applied to distinguish metabolic profiles between the cryptorchidism group and control group. As shown in Figure 1B, clear separation was observed between the two groups, indicating distinct metabolic differences in seminal plasma between the cryptorchidism and the control group, with good within-group consistency. The stability and predictive power of the OPLS−DA model were validated by permutation tests (Figure 1C), confirming the reliability of the discriminative results.

Subsequently, differential metabolites were identified using the criteria of variable importance in projection (VIP) > 1 from multivariate statistical analysis and *p* value < 0.05. A total of 931 differential metabolites were detected, including 486 upregulated and 445 downregulated compounds, with detailed information summarized in Supplementary Table S1. A volcano plot visualized the distribution of these differential metabolites (Figure 1D), and classification analysis revealed that they were mainly categorized into four classes: lipids and lipid-like molecules (296, 40.66%), organic acids and their derivatives (124, 17.03%), organoheterocyclic compounds (86, 11.81%), and benzene derivatives (66, 9.07%) (Figure 1E).

KEGG pathway enrichment analysis was conducted on the differential metabolites, and the top-30 enriched pathways are shown in Figure 1F. Furthermore, the importance of each matched metabolite and pathway was quantified using outdegree centrality. Notably, lipid metabolism and amino acid metabolism pathways were identified as the most significantly perturbed and functionally crucial pathways, specifically including linoleic acid metabolism, sphingolipid metabolism, glycerophospholipid metabolism, the biosynthesis of unsaturated fatty acids, arachidonic acid metabolism, D-amino acid metabolism, and lysine degradation (Figure 1G).

### 3.3. Identification of Seminal Plasma Metabolic Biomarkers in Cryptorchidism with Azoospermia

**Figure 2:**
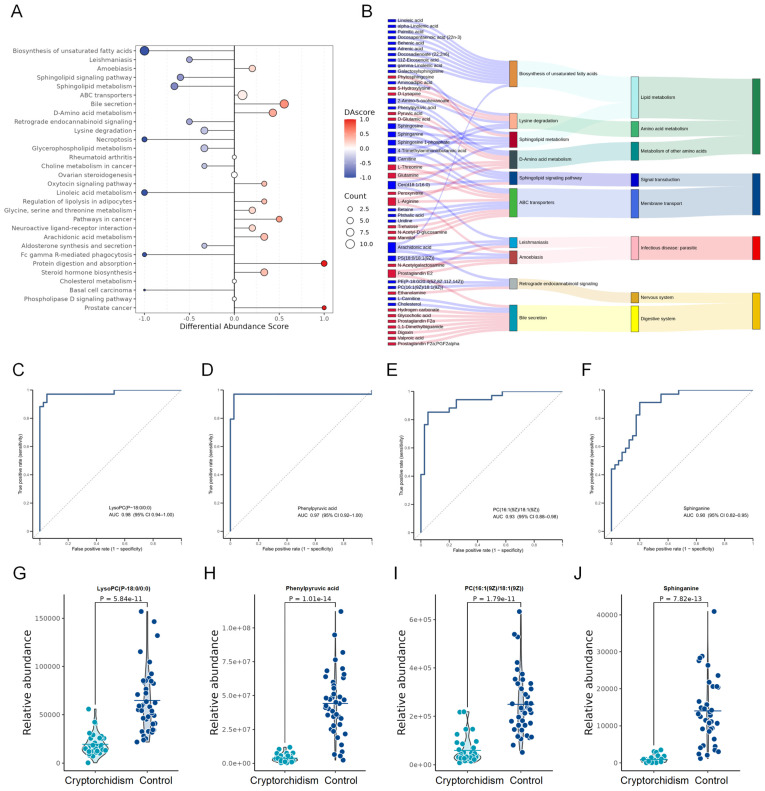
Identification of metabolic biomarkers distinguishing cryptorchidism with azoospermia from control (**A**) Differential abundance (DA) score Plot of Metabolic Pathways, DA score illustrates the overall upregulation/downregulation trend of metabolic pathways by showing the proportion of significantly differential metabolites that are upregulated or downregulated. (**B**) The Sankey diagram illustrates the relationship between the top 10 pathways with significant enrichment and differential metabolites. From left to right, the components are differential metabolites (red indicates upregulation, blue indicates downregulation) and significantly enriched metabolic pathways. (**C**–**F**) Receiver operating characteristic (ROC) curve for the Differential Metabolites. (**G**–**J**) Violin Plot showing the abundance of individual biomarkers in the cryptorchidism with azoospermia and control groups. Statistical significance was assessed by Student’s *t*-test.

To systematically investigate the global trends of metabolic pathway alterations, DA score calculation was performed. As illustrated in Figure 2A, metabolites in most lipid metabolism pathways (Linoleic acid metabolism, Sphingolipid metabolism, Glycerophospholipid metabolism, and Biosynthesis of unsaturated fatty acids) exhibited a global downregulation, whereas Arachidonic acid metabolism was the only lipid pathway with an overall increase. Among amino acid metabolism pathways, metabolites in D-Amino acid metabolism were increased, while those in Lysine degradation were decreased (Figure 2A). Sankey diagram was constructed to delineate the specific upregulated and downregulated metabolites within each key pathway (Figure 2B). Consistent with the DA score patterns, the majority of lipid metabolites were downregulated in patients with cryptorchidism, including Sphingosine, phosphatidylcholine (16:1 (9Z)/18:1 (9Z)), Linoleic acid, phosphatidylserine (18:0/18:1 (9Z)), Arachidonic acid, and other unsaturated fatty acids. In contrast, Prostaglandin E2 was upregulated, consistent with possible inflammatory responses in cryptorchid testes. For amino acid metabolism pathways, D-glutamic acid, pyruvic acid, L-threonine, glutamine, and L-arginine were all increased (Figure 2B).

To identify potential diagnostic biomarkers for cryptorchidism with azoospermia, receiver operating characteristic (ROC) analysis was performed on all differential metabolites. Four top-performing metabolites were selected: LysoPC (P-18:0/0:0) (lysophosphatidylcholine, AUC = 0.98), Phenylpyruvic acid (AUC = 0.97), PC (16:1 (9Z)/18:1 (9Z)) (phosphatidylcholine, AUC = 0.93), and Sphinganine (AUC = 0.90) (Figure 2C–F). All four metabolites displayed significant differences in expression levels between the cryptorchidism and control group (Figure 2G–J), highlighting their potential as candidate biomarkers for cryptorchidism with azoospermia.

### 3.4. Seminal Plasma Metabolic Characteristics of Patients with Successful Versus Failed Sperm Retrieval

Medical records of 35 patients with cryptorchidism were reviewed. Among them 21 patients underwent micro-TESE: 11 cases (52.4%) successfully retrieved sperm including 6 cases of unilateral and 5 cases of bilateral cryptorchidism, while 10 cases (47.6%) failed all of which were bilateral cryptorchidism. Demographic and clinical characteristics of the two surgically treated groups are summarized in Table 2. There were no significant differences observed in mean age, BMI, left/right testicular volume, or interval time of orchidopexy between the successful and failed groups. Hormone profiles also showed no statistically significant differences. Although FSH levels tended to be higher in patients with negative sperm retrieval, the difference did not reach statistical significance. Levels of LH, PRL, testosterone, and E2 were likewise comparable between the two groups (all *p* > 0.05).

**Figure 3:**
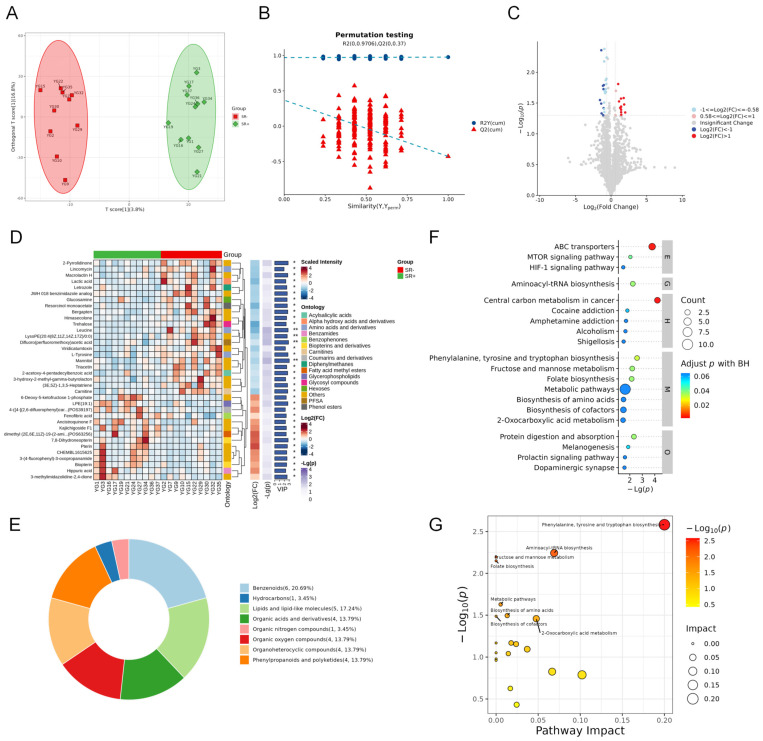
Seminal Plasma Metabolic Characteristics of Patients with Successful and Failed Sperm Retrieval (**A**) OPLS-DA score plot of seminal plasma metabolomic profiles from patients with SR+ (*n* = 11) and SR− (*n* = 10). (**B**) Permutation Test Plot of OPLS−DA. (**C**) Volcano plot of significantly altered metabolites, where colors correspond to Fold Change (FC) and the size of dots corresponds to variable importance in projection (VIP) values from the OPLS−DA model. (**D**) The heatmap of hierarchical clustering analysis of SR+ and SR− group patients. (**E**) Differential metabolites classification ring chart (HMDB Super Class). (**F**) KEGG pathway enrichment bubble plot of differential metabolites. (**G**) Pathway impact plot of KEGG (outdegree centrality). Abbreviations: SR+, sperm retrieval success; SR−, sperm retrieval failure.* *p* < 0.05 and ** *p* < 0.01.

To further explore the metabolic characteristics associated with spermatogenic disorders in the two groups, seminal plasma metabolomic data from both the successful and failed groups were analyzed. The OPLS-DA model showed a clear separation between the two groups (Figure 3A), and the robustness of the model was confirmed by permutation tests (Figure 3B). We subsequently sought to identify differential metabolites between the two groups using the limited sample data, total of 36 differential metabolites was detected, including 14 upregulated and 22 downregulated compounds. A volcano plot visualized the distribution of these differential metabolites (Figure 3C), and heatmap displayed the specific differential metabolites (Figure 3D), with detailed results summarized in Supplementary Table S2. These metabolites were mainly categorized into several major classes: benzene derivatives (6, 20.69%), lipids and lipid-like molecules (5, 17.24%), organic acids and their derivatives (4, 13.79%), and organoheterocyclic compounds (4, 13.79%) (Figure 3E).

KEGG pathway enrichment analysis was performed on these differential metabolites, and the significant pathways was shown in Figure 3F. Furthermore, the importance of each matched metabolite and pathway was calculated using out degree centrality. The results indicated that the significantly abnormal and functionally crucial metabolic pathways mainly included Phenylalanine, tyrosine and tryptophan biosynthesis, Aminoacyl-tRNA biosynthesis, and 2-Oxocarboxylic acid metabolism (Figure 3G).

### 3.5. Seminal Plasma Metabolic Biomarkers Associated with Successful Sperm Retrieval

**Figure 4:**
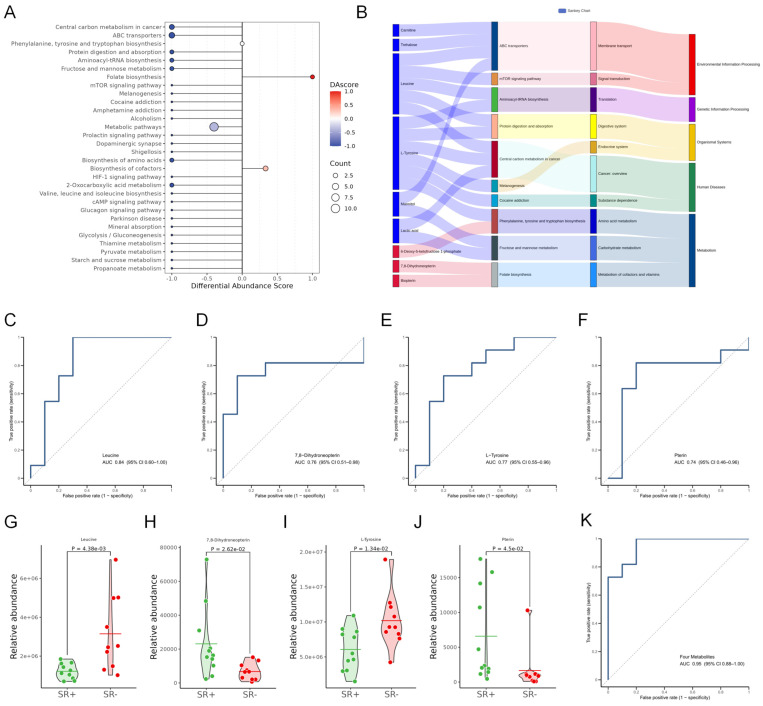
Identification of metabolic biomarkers distinguishing successful from failed sperm retrieval patients. (**A**) Differential abundance (DA) score plot of metabolic pathways; DA score illustrates the overall upregulation/downregulation trend of metabolic pathways by showing the proportion of significantly differential metabolites that are upregulated or downregulated. (**B**) The Sankey diagram illustrates the relationship between the top–10 pathways with significant enrichment and differential metabolites. From left to right, the components are differential metabolites (red indicates upregulation, blue indicates downregulation) and significantly enriched metabolic pathways. (**C**–**F**) Receiver operating characteristic (ROC) curve for the differential metabolites. (**G**–**J**) Violin plot showing the abundance of individual biomarkers in the successful and failed sperm retrieval groups. Statistical significance was assessed by Student’s *t* test. (**K**) ROC curve of the multivariate logistic regression model for the four differential metabolites. Abbreviations: SR+, sperm retrieval success; SR−, sperm retrieval failure.

To investigate the changes in seminal plasma metabolites between the two groups, global trend analysis was conducted based on DA score calculation. As shown in Figure 4A, most metabolites exhibited decreased metabolite levels in the successful sperm retrieval group, while folate biosynthesis was the only pathway demonstrating an overall increase. A Sankey diagram was further constructed to delineate the specific upregulated and downregulated metabolites in each pathway (Figure 4B). The results revealed decreased levels of carnitine, trehalose, leucine, L-tyrosine, mannitol, and lactic acid, whereas increased levels of 6-deoxy-5-ketofructose 1-phosphate, 7,8-dihydroneopterin, and biopterin were observed.

Furthermore, for the exploratory identification of potential biomarkers in seminal plasma from patients who achieved successful sperm retrieval patients, the ROC analysis was performed on each differential metabolite. Four biomarkers demonstrated the highest diagnostic performance: leucine (AUC = 0.84), 7,8-dihydroneopterin (AUC = 0.76), L-tyrosine (AUC = 0.77) and pterin (AUC = 0.74) (Figure 4C–F). All four metabolites showed significant differences between the successful and failed groups (Figure 4G–J). Finally, the multivariate logistic regression model incorporating the four selected metabolites exhibited considerable predictive performance for successful sperm retrieval, yielding an AUC of 0.95 (95% CI: 0.88–1.00) (Figure 4K).

## 4. Discussion

Cryptorchidism with azoospermia remains a major clinical challenge, both in understanding its pathogenesis and in predicting sperm retrieval outcomes. Here, we performed a comprehensive metabolomic analysis of seminal plasma from cryptorchidism with azoospermia and healthy controls, and further investigated metabolic differences between patients with successful and failed micro-TESE outcomes. Our findings reveal that cryptorchidism with azoospermia is characterized by widespread metabolic dysregulation, particularly in lipid and amino acid metabolism. Notably, we identified a distinct panel of metabolites that not only differentiate cryptorchidism from healthy controls but also correlate with spermatogenic potential, providing predictive value for successful sperm retrieval in micro-TESE.

Compared with normal controls, cryptorchidism with azoospermia exhibits significant metabolic abnormalities in seminal plasma, with 931 differential metabolites enriched in lipid metabolism and amino acid metabolism pathways, which is consistent with their critical roles in spermatogenesis [23]. Lipids are the main components of sperm membranes and the primary energy source for sperm motility [24]. The present study found that most lipid metabolism pathways are downregulated in cryptorchidism patients, leading to reduced levels of key metabolites such as sphingosine, linoleic acid, and phosphatidylcholine. As an essential ω-6 unsaturated fatty acid in humans, linoleic acid is crucial for maintaining sperm membrane fluidity and flexibility; its reduction may directly impair sperm flagellar motility and membrane integrity, thereby contributing to spermatogenic failure [25]. Sphingolipids and glycerophospholipids are key structural components of the sperm acrosome and tail, and their deficiency disrupts the acrosome reaction and sperm motility—all these results are consistent with the azoospermic phenotype of cryptorchidism [26,27].

Notably, arachidonic acid metabolism is the only upregulated lipid pathway in the seminal plasma of cryptorchidism patients, with elevated levels of prostaglandin E2. Arachidonic acid derivatives (e.g., prostaglandins, leukotrienes) are potent inflammatory mediators. The increase in prostaglandin E2 is consistent with possible inflammatory responses in the testicular microenvironment of cryptorchidism patients. Chronic testicular inflammation can induce spermatogenic cell apoptosis through the excessive production of reactive oxygen species and release of pro-inflammatory cytokines, further exacerbating spermatogenic dysfunction [28]. This finding supports the critical role of inflammation in the pathogenesis of cryptorchidism, which may be associated with the long-term exposure of the testes to abdominal hyperthermia and oxidative stress.

Amino acids are vital for sperm protein synthesis, energy metabolism, and oxidative stress defense. The present study showed that the D-amino acid metabolism pathway is upregulated, while the lysine degradation pathway is downregulated in cryptorchidism patients. D-amino acids can regulate sperm motility and acrosome reaction by modulating ion channels on the sperm membrane [29]. However, the excessive accumulation of D-glutamic acid may disrupt the acid–base balance of seminal plasma, indirectly affecting sperm survival. Downregulation of the lysine degradation pathway reduces energy supply from amino acid catabolism and impairs ammonia detoxification, which may be one of the causes of spermatogenic arrest in cryptorchidism patients [30].

We identified four metabolites with high diagnostic efficacy for azoospermia with cryptorchidism: LysoPC (P-18:0/0:0), phenylpyruvic acid, PC (16:1 (9Z)/18:1 (9Z)), and sphinganine, with AUC values all > 0.91. LysoPC and PC are key components of sperm membranes, and their abnormal levels directly reflect membrane structural damage, serving as direct indicators of impaired spermatogenic function [31]. Phenylpyruvic acid, a metabolite of phenylalanine degradation, is associated with oxidative stress and amino acid metabolism disorders [32]. Sphinganine, a precursor of sphingolipids, is critical for sperm membrane synthesis and cell signaling [33]. Collectively, these metabolites constitute potential diagnostic indicators for cryptorchidism with azoospermia, overcoming the limitations of traditional markers.

To our knowledge, this study is among the first to explore metabolic differences between cryptorchidism with azoospermia patients with successful and failed micro-TESE. Our exploratory identification of 36 differential metabolites and key pathways provides important clues for predicting residual spermatogenic potential in the testes of cryptorchidism with azoospermia patients. Notably, folate biosynthesis is the only upregulated pathway in the successful group, with elevated levels of 7,8-dihydroneopterin and biopterin. Folate is an essential substance for DNA synthesis, methylation, and repair—processes crucial for the proliferation and differentiation of spermatogonial stem cells [34,35]. Upregulated folate biosynthesis improves DNA replication fidelity, reduces DNA damage, and supports the survival of residual spermatogenic cells. As a cofactor for nitric oxide synthase, biopterin also regulates testicular blood flow and maintains the stability of the spermatogenic microenvironment, further promoting spermatogenesis [36]. This finding suggests that cryptorchidism patients with residual spermatogenic function retain the ability to upregulate folate biosynthesis to protect spermatogenic cells from damage, which may be the underlying reason for their successful sperm retrieval.

The phenylalanine, tyrosine and tryptophan biosynthesis and aminoacyl-tRNA biosynthesis pathways were significantly perturbed between the successful and failed groups. Tyrosine, a product of this pathway, is a precursor for dopamine and testosterone synthesis; its reduced levels in the successful group may reflect the spermatogenic potential of the testis [37]. Aminoacyl-tRNA biosynthesis is a key step in protein synthesis, and its downregulation in the successful group may indicate a moderate protein synthesis rate that supports limited sperm production [38,39]. For predicting micro-TESE outcomes, four metabolites—leucine, 7,8-dihydroneopterin, L-tyrosine and pterin—exhibited moderate predictive value. Finally, the multivariate logistic regression model incorporating the four selected metabolites exhibited considerable predictive performance for successful sperm retrieval, yielding an AUC of 0.95. Notably, no significant differences in these metabolites were observed between the cryptorchidism and control groups, indicating they are indicators specifically associated with residual spermatogenic potential rather than general cryptorchidism-related metabolic abnormalities—making them ideal predictive biomarkers.

This study has several limitations. First, the sample sizes of the successful and failed sperm retrieval groups are relatively small, and multi-center, large-cohort studies are required to validate the predictive value of the candidate biomarkers. Second, untargeted metabolomics only provides preliminary metabolic profiles; a quantitative analysis of key metabolites and a confirmation of their expression patterns are highly necessary as a next step to be performed via targeted metabolomics. Third, this study focuses on metabolic phenotypes, and mechanistic studies such as in vitro cell models or animal experiments are necessary to clarify the specific processes by which these metabolic perturbations drive the pathogenesis of cryptorchidism.

## 5. Conclusions

In summary, the present study demonstrates that patients with cryptorchidism and azoospermia exhibit extensive metabolic perturbations in seminal plasma, particularly involving lipid metabolism, amino acid metabolism, and inflammation-related pathways. Notably, the folate biosynthesis pathway and specific differential metabolites appear to play a crucial role in preserving residual spermatogenic function and predicting micro-TESE sperm retrieval outcomes of micro-TESE. Our exploratory identified candidate biomarkers provide promising tools for the clinical diagnosis of cryptorchidism and the prediction of sperm retrieval outcomes, which is conducive to optimizing the treatment strategies for patients with cryptorchidism. These findings deepen our understanding of the pathogenic mechanisms underlying cryptorchidism and offer new avenues for clinical management.

## Figures and Tables

**Table 1:** Characteristics of participants.

	Cryptorchidism (*n* = 35)	Control (*n* = 40)	*p* Value
Age (year)	32.0 ± 4.4	30.5 ± 4.9	0.3170
BMI (kg/m^2^)	26.0 ± 3.9	24.9 ± 4.9	0.7021
Left testis volume (mL)	8.4 ± 4.3	14.3 ± 1.3	0.0003
Right testis volume (mL)	9.1 ± 5.6	14.7 ± 1.5	0.0094
PRL (mIU/L)	14.6 ± 9.7	11.2 ± 5.9	0.3617
FSH (IU/L)	19.7 ± 13.5	5.6 ± 2.2	0.0034
LH (IU/L)	9.1 ± 5.5	4.0 ± 1.8	0.0085
Testosterone (ng/mL)	12.6 ± 13.4	11.2 ± 5.1	0.7844
E2 (pg/mL)	125.7 ± 93.4	103.6 ± 28.6	0.5210

Note: Data are presented as mean ± SD. PRL, prolactin; FSH, follicle-stimulating hormone; LH, luteinizing hormone; E2, estradiol.

**Table 2:** Characteristics of participants in micro-TESE surgery.

	Success (*n* = 11)	Failure (*n* = 10)	*p*-Value
Age (year)	31.3 ± 3.9	33.5 ± 4.2	0.2553
BMI (kg/m^2^)	25.8 ± 5.1	26.2 ± 2.4	0.8707
Left testis volume (mL)	9.7 ± 4.3	7.2 ± 3.0	0.1407
Right testis volume (mL)	10.1 ± 5.7	7.1 ± 3.1	0.1678
Unilaterality	6	0	<0.0001
Bilaterality	5	10	0.0044
Orchidopexy interval (year)	17.3 ± 5.2	14.6 ± 6.5	0.0782
PRL (mIU/L)	13.4 ± 9.2	11.5 ± 7.5	0.6577
FSH (IU/L)	13.1 ± 9.8	22.0 ± 15.7	0.1275
LH (IU/L)	6.8 ± 5.1	8.9 ± 4.6	0.3239
Testosterone (ng/mL)	9.7 ± 6.3	7.3 ± 5.8	0.2919
E2 (pg/mL)	120.1 ± 87.2	93.6 ± 56.1	0.4744

Note: Data are presented as mean + SD. PRL, Prolactin; FSH, follicle-stimulating hormone; LH, luteinizing hormone; E2, Estradiol.

## Data Availability

The original contributions presented in this study are included in the article. Further inquiries can be directed to the corresponding authors.

## Supplementary Materials

The following supporting information can be downloaded at: https://www.mdpi.com/article/10.3390/metabo16020147/s1, Table S1: Differential metabolites between CR and NC; Table S2: Differential metabolites between SR+ and SR−.
